# Nationwide Longitudinal Analysis of Acute Liver Failure in Taiwan

**DOI:** 10.1097/MD.0000000000000035

**Published:** 2014-05-02

**Authors:** Cheng-Maw Ho, Chih-Hsin Lee, Jann-Yuan Wang, Po-Huang Lee, Hong-Shiee Lai, Rey-Heng Hu

**Affiliations:** Department of Surgery (CMH, PHL, HSL, RHH), Department of Internal Medicine (JYW), National Taiwan University Hospital, Graduate Institute of Clinical Medicine (CMH, PHL, HSL), and Department of Internal Medicine (JYW), College of Medicine, National Taiwan University, Department of Pulmonary Medicine (CHL), Wanfang Hospital, Taipei Medical University, Taipei, Taiwan.

## Abstract

Acute liver failure (ALF) is uncommon but fatal. Current management is based mostly on clinical experience. We aimed to investigate the incidence, etiology, outcomes, and prognostic factors of ALF in Taiwan. Patients with the admission diagnosis of ALF between January 2005 and September 2007 were identified from the Longitudinal Health Insurance Database of Taiwan. ALF was further confirmed by disease severity based on laboratory orders, prescriptions, and duration of hospital stay, and acute onset without prior liver disease. Prognostic factors were identified using Cox regression analysis. During the study period, 218 eligible cases were identified from 28,078 potential eligible ALF patients. The incidence was 80.2 per million person-years in average and increased with age. The mean age was 57.9 ± 17.1 years and median survival was 171 days. The most common etiologies were viral (45.4%, mainly hepatitis B virus) and followed by alcohol/toxin (33.0%). Independent prognostic factors included alcohol consumption (hazard ratio, HR, 1.67 [1.01–2.77]), malignancy (HR 2.90 [1.92–4.37]), frequency of checkups per week for total bilirubin (HR 1.57 [1.40–1.76]), sepsis (HR 1.85 [1.20–2.85]), and the use of hemodialysis/hemofiltration (HR 2.12 [1.15–3.9]) and proton pump inhibitor (HR 0.94 [0.90–0.98]). Among the 130 patients who survived ≥90 days, 66 (50.8%) were complicated by liver cirrhosis. Eight (3.7%) were referred for liver transplantation evaluation, but only 1 received transplantation and survived. ALF in Taiwan is mainly due to viral infection. Patients with malignancy and alcohol exposure have worst prognosis. The use of proton pump inhibitor is associated with improved survival. Half of the ALF survivors have liver cirrhosis.

## INTRODUCTION

Acute liver failure (ALF) is an uncommon clinical syndrome that often has a course associated with rapidly progressive multiorgan failure and devastating complications like coagulopathy and encephalopathy in patients without previous liver disease. Its etiologies include a multitude of infectious, immunologic, infiltrative, or metabolic diseases, and have considerable geographical and ethnic variations.^1^ In developing countries, viral causes predominate, whereas drugs or toxins are recognized as common causes in the United States and the United Kingdom.^1^ Reports estimate an overall incidence of fewer than 10 cases per million persons per year in developed countries.^2–4^ Because ALF is an orphan disease, large clinical trials are extremely difficult and its management is currently based on clinical experience rather than on solid evidence.^2,5,6^ Conclusions are also very difficult to reach even in a systemic review^7^ because of the varying definitions of ALF among studies. As such, mortality rate remains high at 60% to 80%.^8^

The most widely accepted definition of ALF includes evidence of coagulation abnormality and a degree of mental alteration (encephalopathy) in a patient without preexisting liver disease.^1,5^ No single institute has established considerable case series except King’s College Hospital,^9^ whereas most currently available reports are multicenter collaborations.^4,10–14^

As the mandatory universal health insurance program offering comprehensive medical care coverage, the NHI of Taiwan has covered up to 99% of residents in Taiwan for several years since 1996.^15^ With a longitudinal follow-up of more than 20 million subjects and validated diagnoses of catastrophic illness,^16,17^ the National Health Insurance Research Database (NHIRD) provides a very suitable research material to explore the outcome of a rare disease or clinical entity. The aim of this study is to analyze the incidence, characteristics, hospital course, prognosis, and complications of ALF in Taiwan using the longitudinal cohort information of the NHIRD.

## PATIENTS AND METHODS

The institutional review board of National Taiwan University Hospital, Taipei, Taiwan approved this study (NTUH REC: 201212001W). As a retrospective study using an encrypted database, the institutional review board waived the need for informed consent.

### Data Source

The Longitudinal Health Insurance Database (LHID) 2005, a subset database of the NHI program, contains the entire original claim data from 1996 to 2007 of 1,000,000 beneficiaries randomly sampled from the 2005 Registry for Beneficiaries of the NHI program.

### Patient Selection

From the LHID 2005, patients who were admitted due to ALF for the first time from January 1, 2005 to September 30, 2007 were identified. Patients with possible ALF were identified based on inpatient records with compatible diagnoses, laboratory orders for ammonia and international normalized ratio (INR), and prescription of lactulose (regardless of the route of administration) and stayed in hospital for ≥7 days to ensure severe liver injury. The compatible diagnoses of ALF included International Classification of Diseases, Ninth Revision, Clinical Modification (ICD-9-CM) code for ALF (570.0), hepatic coma (472.2), autoimmune hepatitis (571.42), acute alcoholic hepatitis (571.1), hepatitis unspecified (573.3), jaundice (782.4), viral hepatitis (070.0–070.9), and hepatitis B carrier (V02.61).

To ensure no preexisting liver disease, patients were excluded if they had any of the following diagnoses within 3 years prior to the index admission: chronic hepatitis (ICD-9-CM 571.4), hepatic stone (ICD-9-CM 574.5), hepatocellular carcinoma (ICD-9-CM 155.0), intrahepatic cholangiocarcinoma (ICD-9-CM 155.1), gall bladder cancer (ICD-9-CM 156.0), extrahepatic bile duct cancer (ICD-9-CM 156.1), malignant neoplasm of the pancreas or ampulla of Vater (ICD-9-CM 157.9, 156.2), liver metastasis (ICD-9-CM 197.7), and liver cirrhosis (ICD-9-CM 571.2, 571.5, 571.6). Those admitted after October 1, 2007 were excluded to ensure a minimal follow-up duration of 3 months.

For every enrolled patient, the demographic data, laboratory tests, medications, clinical procedures, and outcomes were retrieved from the LHID 2005 and the possible etiology of acute hepatic failure was determined.

### Demographic Data

Demographic information including sex, age, underlying comorbidity (ie, diabetes mellitus, chronic obstructive pulmonary lung disease, end-stage renal disease, autoimmune disorder, acquired immune deficiency syndrome, and malignancy), and low income were collected as in a previous report.^18^

### Laboratory Tests, Medications, and Procedures

The frequency of laboratory tests, including INR, total bilirubin, direct bilirubin, aspartate aminotransferase, alanine aminotransferase, and ammonia were calculated. Prescriptions of medications, including lactulose, diuretics, vasopressin (glypressin, somatosatin, and sandostatin), and proton pump inhibitors (PPIs), were converted from the claims data according to the defined daily doses and grouped according to their pharmacologic categories.^19^ The performance of procedures (intubation for ventilator, plasmapheresis, hemodialysis, hemofiltration, upper gastrointestinal [GI] panendoscopy, echo-guided fluid tapping, and blood transfusion), transplantation-associated laboratory test (human leukocyte antigen), or liver transplantation (LT) procedure were recorded. Transfusion of fresh frozen plasma >30 units in a week or >100 units during the whole course of index admissions were considered as plasmapheresis.

### Etiologic Contribution

The etiology of ALF was based on the priority of virus infection, alcohol, and metabolic causes, and then hepatotoxin (see descriptions in the Supplemental Digital Content 1 for potential hepatotoxins, http://links.lww.com/MD/MD-D-14-00079), if ever. Malignant infiltration was attributed to the presence of malignancy and the lack of the etiologies mentioned above.

### Follow-Up and Outcome

The patients were followed-up until death, withdrawal of health insurance, or December 31, 2007. The date of death was obtained from the cause-of-death data included in the LHID 2005. Based on the discharge diagnosis (ICD-9-CM), intrahospital complications were noted, including hemorrhage (GI tract: 578.9, 531.4, 532.4, 530.82; brain: 431, 432.0, 432.9, 852.0–4, 767.0, 772.2; unspecified: 459.0), sepsis (995.91, 995.92), pneumonia (481, 482, 484, 486), extrahepatic organ damage (renal insufficiency: 584.5–9, 572.4; respiratory failure: 518.81, 518.84, 786.0, 799.1), and seizure (345.0–4).

### Statistical Analysis

Data were expressed as mean ± standard deviation, median (interquartile range [IQR]), or number (percentage) when appropriate. The Student *t*-test or χ^2^ test was used for intergroup comparison. The survival curves of different etiologic groups were generated using the Kaplan–Meier method and compared using the log-rank test. The Cox proportional hazard model was used to identify independent prognostic factors. The *P* value in each variable was derived from the Wald test in the Cox model and was used to predict and identify independent prognostic factors. Sensitivity analyses were further performed in the subpopulation that had no concomitant malignancy, because it was difficult to attribute the etiology of ALF accurately in patients with concomitant malignancy. Risk factors for intrahospital complications were analyzed using logistic regression analysis. A 2-sided *P* < 0.05 was considered significant. All analyses were performed with the Statistical Package for the Social Sciences version 18.0 (IBM Corporation, Armonk, NY).

## RESULTS

### Demographic Characteristics of the ALF Cohort

**FIGURE 1:**
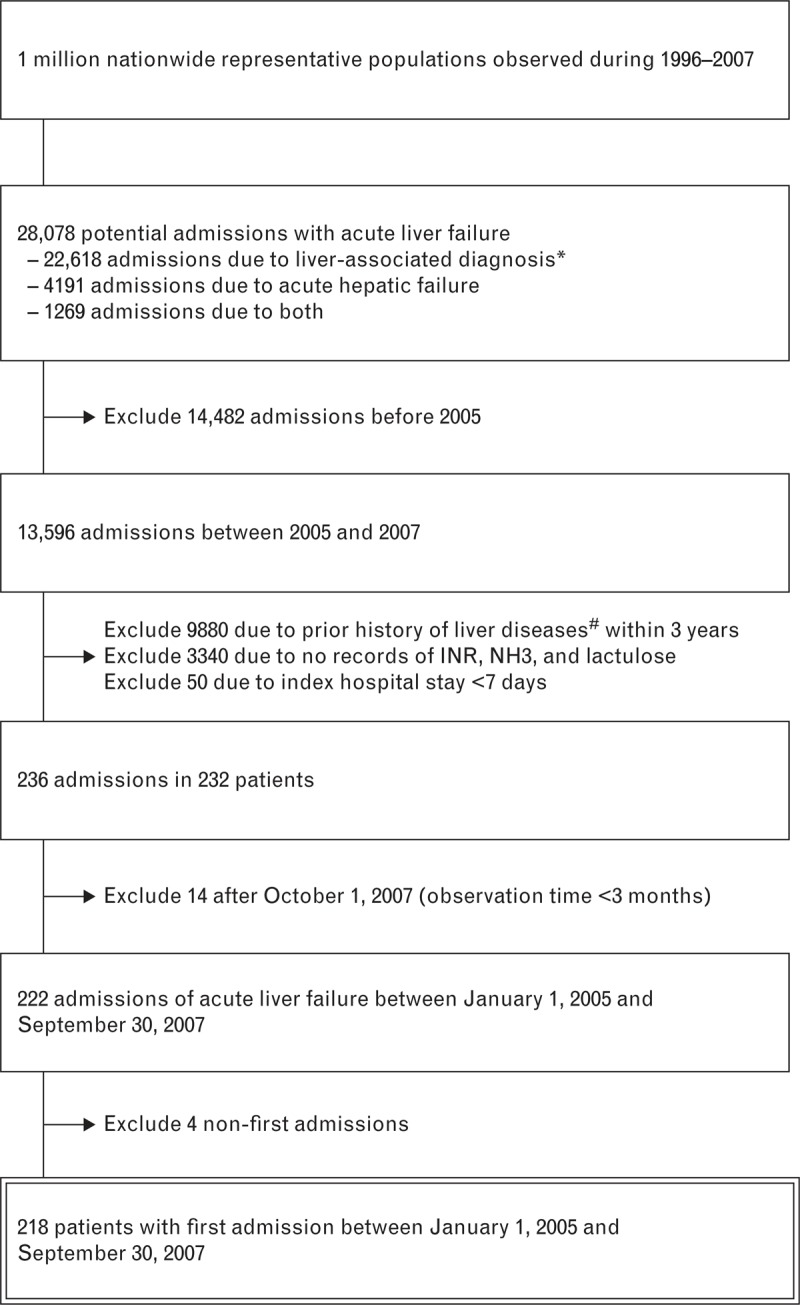
Schematic representation of the patient selection process. INR = international normalized ratio, NH3 = ammonia, TB = tuberculosis. *Liver-associated diagnosis included International Classification of Diseases, Ninth Revision, Clinical Modification (ICD-9-CM) 070.0–070.9, 571.1, 571.42, 573.3, 782.4, and V02.61. ^#^Prior history of liver diseases included liver-associated diagnoses; chronic hepatitis (ICD-9: 571.4); hepatic stone (ICD-9: 574.5); hepatocellular carcinoma (ICD-9: 155.0); intrahepatic cholangiocarcinoma (ICD-9: 155.1); malignant neoplasm of gall bladder (ICD-9: 156.0); malignant neoplasm of extrahepatic bile ducts (ICD-9: 156.1); malignant neoplasm of ampulla of Vater (ICD-9: 156.2); malignant neoplasm of pancreas (ICD-9: 157.9); and liver metastasis (ICD-9: 197.7).

A total of 28,078 potentially eligible admissions for acute liver failure were identified from the 2,719,680.2 person-years of follow-up since 2005 in LHID 2005 (Figure 1). Among them, 14,482 admissions before 2005 were excluded, as well as 9880 with prior history of liver diseases within 3 years, 3430 without records of INR, ammonia, or lactulose, and 50 with hospital stay less than 7 days. Another 14 admissions were excluded to guarantee an observation time ≥3 months. Four were also excluded due to non-first admissions. The remaining 218 patients were enrolled in this study.

**FIGURE 2:**
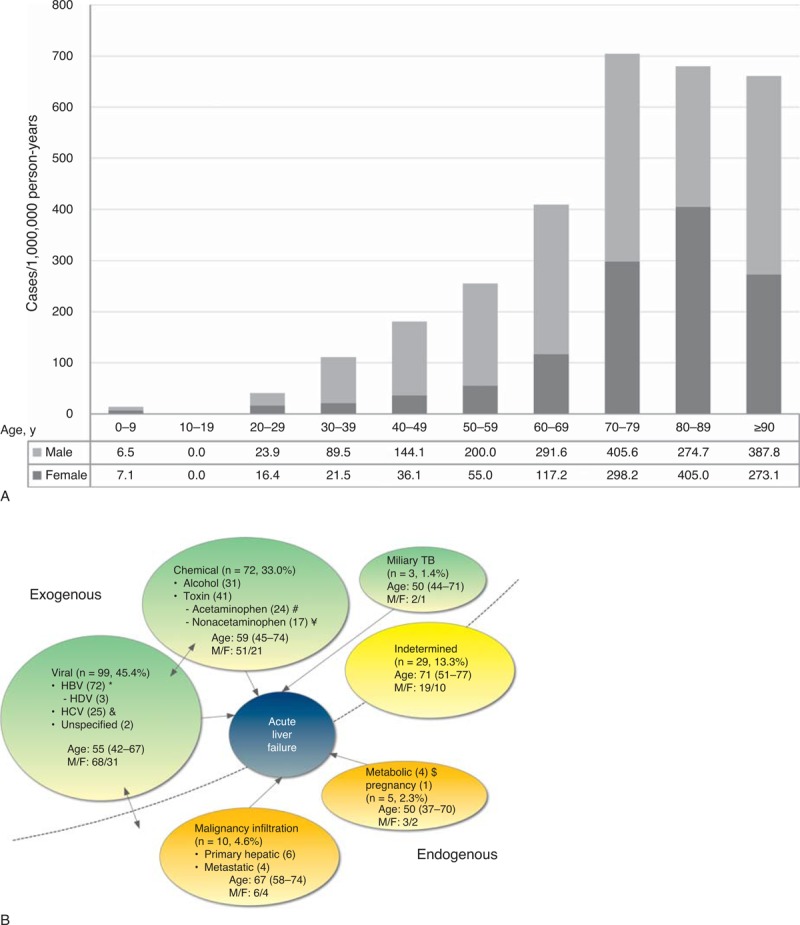
The calculated incidence (A) and etiology (B) of acute liver failure in Taiwan. (A) Note that the incidence increased with age in both genders. HBV = hepatitis B virus, HCV = hepatitis C virus, HDV = hepatitis D virus. *Fourteen were pathologically exposed to alcohol, 5 coinfected with hepatitis C virus, 2 exposed to TB, and 1 also exposed to recent anti-TB medications and 1 Wilson disease of age 25, and 4 pathologically exposed to alcohol. ¥ 18 were also exposed to nonacetaminophen hepatotoxic agents and 2 exposed to herbal agents. $ 4 were also exposed to herbal agents. £ 1 was exposed to Wilson disease of age 44.

The 218 ALF patients (150 males) had a mean age of 57.9 ± 17.1 years and median age of 57.3 years (range, 45.4–72.5 years). The incidence was 80.2 per million person-years (218 cases in 2,719,680.2 person-years) and this increased with age (Figure 2A). The median follow-up duration was 171 days (range, 7–1059 days). Attributable etiologic exposures were viral infection [45.4%, mainly hepatitis B virus (HBV)], chemicals (alcohol or toxin) (33.0%), infiltrative malignancy (4.6%), miliary tuberculosis (1.4%), and others (metabolic or pregnancy: 2.3%; indeterminate: 13.3%) (Figure 2B).

The primary site of concomitant malignancy and etiologic exposure of ALF patients were listed in Table S1 in the Supplemental Digital Content 2 (http://links.lww.com/MD/MD-D-14-00079). The most common malignancy was hepatocellular carcinoma (63%), followed by colorectal cancer, lung cancer, and head and neck cancer (7% each).

The clinical characteristics were presented in Table 1. Of the 218 patients, 88 (40.4%) died within 90 days after admission, with a median survival of 29 (IQR, 7–93) days. Eighty-one (37%) died during their index admission. Among the 130 patients who survived ≥90 days, the median follow-up duration was 458.5 (IQR, 45–1059) days. The former group was statistically significantly older (60.0 vs 56.5 years; *P* = 0.018) and had longer hospital stay (*P* = 0.020), higher probability of intensive care unit (ICU) admission (*P* = 0.018), and higher prevalence of concomitant malignancy (47% vs 14%; *P* < 0.001).

**TABLE 1:**
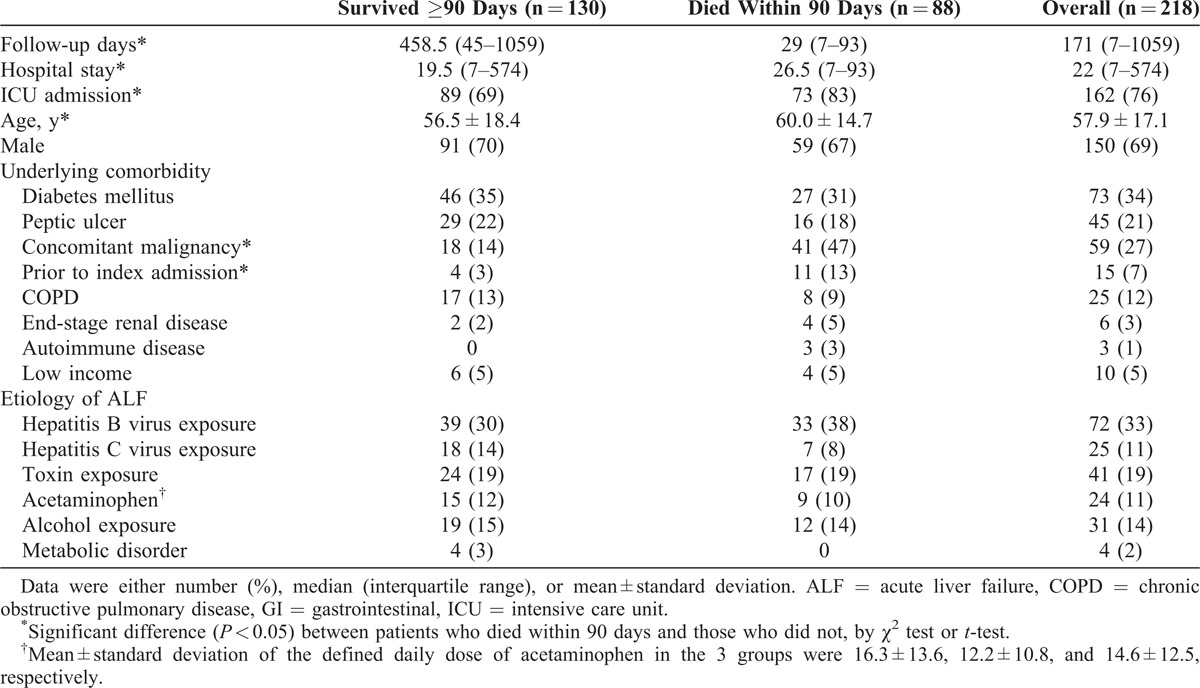
Characteristics of Patients With Acute Liver Failure (ALF)

### Severity and In-Hospital Complications of the ALF Cohort

Compared with those who survived ≥90 days, the patients who died within 90 days after admission received more frequent checkups of total bilirubin (1.9 vs 0.4 per week; *P* < 0.001) and ammonia (1.4 vs 1.0 per week; *P* = 0.008), and were more likely to receive plasmapheresis (15% vs 8%, *P* = 0.045) (Table 2). There were no differences between the 2 groups regarding the presence of ascites and esophageal varices, frequency of checkup for aspartate aminotransferase, alanine aminotransferase, direct bilirubin, and INR levels, and proportion of patients who underwent procedures (ie, panendoscopy, computed tomography, or brain magnetic resonance imaging).

**TABLE 2:**
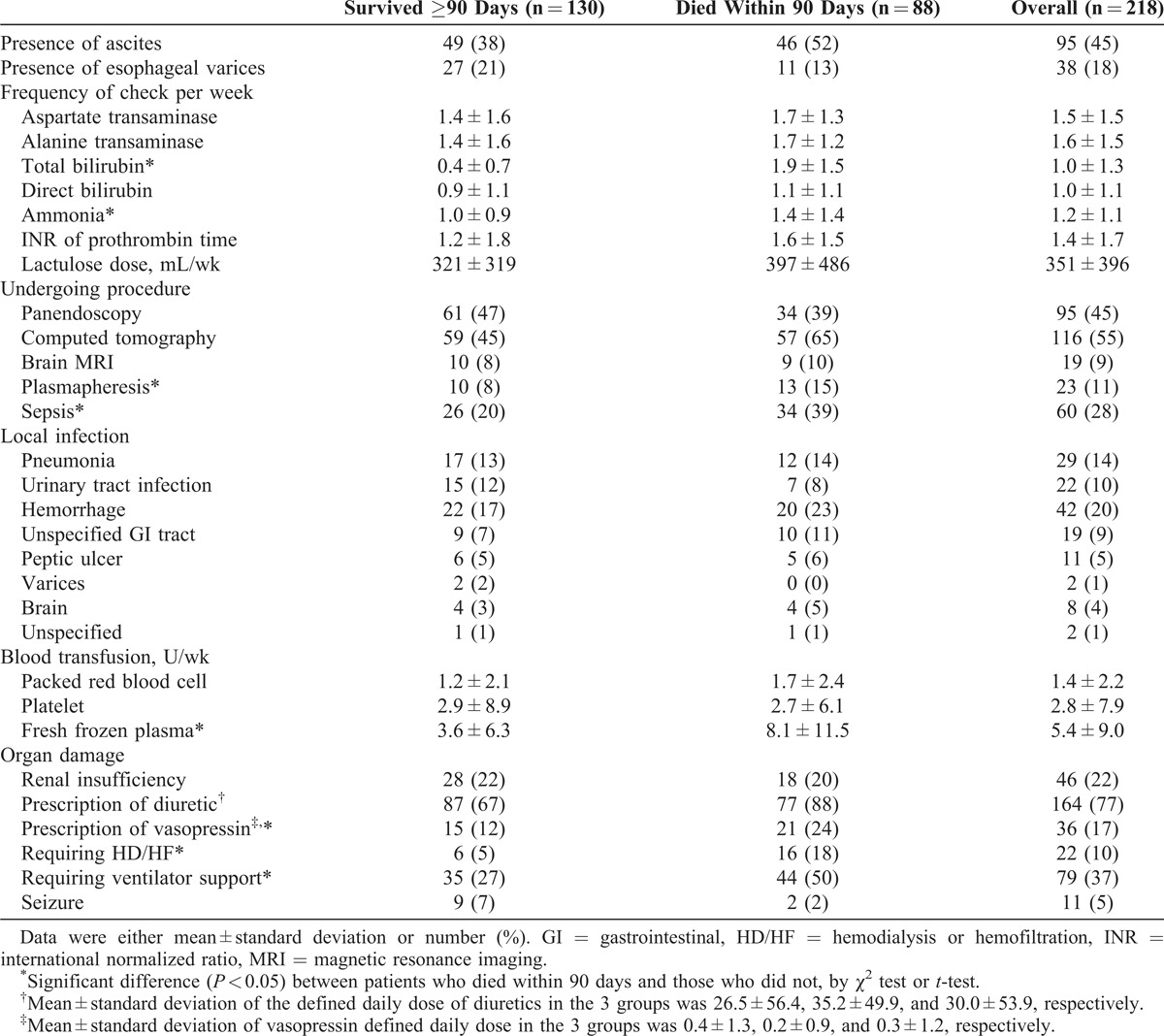
Severity and Complications of Acute Liver Failure During Index Admission

Compared with those who survived ≥90 days, patients who died within 90 days after admission were more likely to be complicated by sepsis (39% vs 20%; *P* = 0.003), required more frequent transfusion of fresh frozen plasma (8.1 vs 3.6 units/wk; *P* < 0.001), use of vasopressin (24% vs 12%; *P* = 0.025), and required renal replacement therapy (18% vs 5%; *P* = 0.001) and ventilator support (50% vs 27%; *P* = 0.001) (Table 2).

Logistic regression analysis revealed that peptic ulcer (hazard ratio [HR] [95% confidence interval] 6.96 [2.87–16.91]; *P* < 0.001) and respiratory failure (HR: 3.20 [1.30–7.85]; *P* = 0.011) were independent risk factors of in-hospital hemorrhage complication. For the occurrence of sepsis, renal insufficiency (HR 2.55 [1.15–5.65]; *P* = 0.021), computed tomography (HR 2.61 [1.27–5.34]; *P* = 0.009), and frequency of checkups per week for total bilirubin (HR 1.35 [1.05–1.72]; *P* = 0.019) were risk factors.

### Long-Term Sequelae

Among the 130 patients who survived ≥90 days after admission, 66 (51%) were complicated by liver cirrhosis, including 22 with encephalopathy, and 21 with ascites. During follow-up, 20 (15%) required vasopressin in subsequent admissions and 29 (22%) underwent panendoscopy. Sixty-four (49%) patients, including 10 without liver cirrhosis, received lactulose whereas 72 (55.4%), including 19 without liver cirrhosis, received diuretics.

### Survival Analysis

The 1- and 2-year survival probabilities were 49.3% and 45.9%, respectively. Eight were referred for LT evaluation. Among them, 3 survived without LT and 1 survived with LT. Kaplan–Meier analysis revealed that the survival of 59 patients with concomitant malignancy were significantly worse than that of the 159 without malignancy (*P* < 0.001) (Figure S1A of the Supplemental Digital Content 3, http://links.lww.com/MD/MD-D-14-00079, which illustrated the survival curves of ALF patients stratified according to the status of concomitant malignancy). In the latter group, the 1- and 2-year survival rates were 61.9% and 57.3%, respectively, and were 14.6% and 14.6%, respectively, in the former group. Among patients without malignancy, the Kaplan–Meier survival curves for different etiologic groups were shown in Figure S1B of the Supplemental Digital Content 3 (http://links.lww.com/MD/MD-D-14-00079).

The results of multivariate Cox regression revealed that in patients with ALF, the independent factors associated with poor survival were alcohol consumption (HR 1.67 [1.01–2.77]; *P* = 0.046), malignancy on index admission (HR 2.90 [1.92–4.37]; *P* < 0.001), frequency of checkups per week for total bilirubin (HR 1.57 [1.40–1.76]; *P* < 0.001), sepsis (HR 1.85 [1.20–2.85]; *P* = 0.005), and use of hemodialysis/hemofiltration (HR 2.12 [1.15–3.9]; *P* = 0.015) and PPIs (HR 0.94 [0.90–0.98]; *P* = 0.005) (Table 3).

**TABLE 3:**
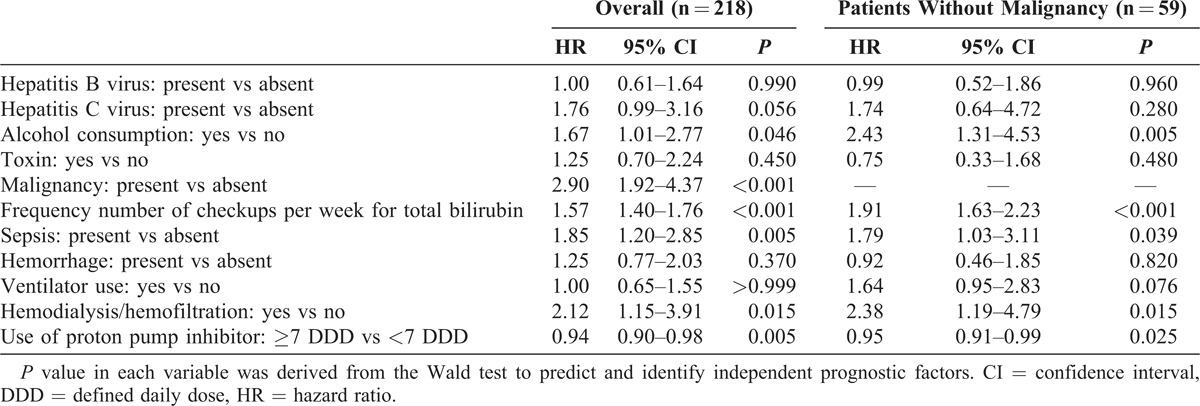
Risk Factors of Survival in All Patients With Acute Liver Failure and in Those Without Malignancy on Index Admission in Cox Proportional Hazard Model

Sensitivity analysis focusing on the subpopulation without malignancy showed that 5 variables—alcohol consumption (HR 2.43 [1.31–4.53]; *P* = 0.005), frequency of checkups per week for total bilirubin (HR 1.91 [1.63–2.23]; *P* < 0.001), sepsis (HR 1.79 [1.03–3.1]; *P* = 0.039), and use of hemodialysis/hemofiltration (HR 2.38 [1.19–4.79]; *P* = 0.015) and PPIs (HR 0.95 [0.91–0.99]; *P* = 0.025)—remained significant prognostic factors (Table 3). Among patients without concomitant malignancy, the adjusted survival curves for patients stratified by etiology of ALF (Figure S1C of the Supplemental Digital Content 3, http://links.lww.com/MD/MD-D-14-00079) demonstrated that alcoholic patients had the worst survival, whereas those with hepatitis C virus or toxin exposures had more favorable outcomes.

## DISCUSSION

Analyzing the nationwide ALF cohort, the present study has 4 main findings. First, the incidence of ALF was 80.2 per million person-years, which increased with age. Second, viral infection was the most common attributable etiology (45.4%). Third, the mortality rate was 40.4% within 90 days after admission and liver cirrhosis occurred in about half of the survivors. Lastly, alcohol consumption, malignancy on index admission, frequency of checkups per week for total bilirubin, sepsis, and use of hemodialysis/hemofiltration and not using PPIs were poor prognostic factors for ALF.

The observation that the incidence of ALF increases with age is interesting. The median age of the 218 ALF patients is 57.3, quite older than the 38 years of a previous study with 308 ALF patients.^10^ In another previous study using the NHIRD (1997–2004) to assess drug-induced liver injury, the age distribution is also skewed towards >60 years.^20^ This may indicate the ageing population of Taiwan. Ageing is accompanied by diminished metabolism and elimination of toxin or alcohol,^21,22^ decreased water distribution volume,^23^ and reduced liver regeneration when the liver is placed under stressful conditions like hepatectomy or acute liver injury.^2,21,23^ Acute liver injury or postischemic liver injury is greater in older adult mice than in younger ones.^24,25^ Older donor age is a well-known risk factor of poorer outcome of liver recipients.^21,26,27^ Ageing may also explain the higher ALF incidence in the study compared with that in literature.^2–4^

In the nationwide cohort, the major etiological exposure of ALF in Taiwan is HBV infection, followed by toxins and alcohol. HBV infection is a leading cause of ALF in Japan and Spain, whereas toxins are more common in the United States, the United Kingdom, and Korea.^1,2,4,6,13,28,29^ Whereas studies conducted in Japan exclude patients with alcohol exposure for analysis,^12,30,31^ some in the United States consider alcohol as playing a contributing but unclear role in ALF.^3,11^ In the current ALF cohort, about one fourth have multiple etiologic exposures, suggesting that their ALF may be attributed to multiple hits in a short period of time. There have been reports showing that acute hepatitis C in patients with concurrent chronic HBV infection is associated with a substantial risk of ALF.^32,33^ It is also clear that genetic polymorphisms, or the effects of concomitant drugs, alcohol, or diseases, can alter the threshold for exposure to other toxic metabolites and result in ALF.^34^

Despite the well-known high mortality rate of ALF, reports on its long-term sequelae are lacking. This study shows that liver cirrhosis occurs in about half of ALF survivors within less than 3 years. Cases of ALF with features suggestive of an autoimmune pathogenesis have higher incidence of chronic hepatitis in long-term follow-up than those without such features.^35^ Acute liver injury, even those caused by single-dose or short-term administration of hepatotoxic agents such as temozolomide, can be followed by prolonged liver damage.^36^ Furthermore, the quality of life is significantly impaired in long-term survivors of ALF.^37^ All of these findings suggest that ALF may have some sustained irreversible impact. As the course of ALF is widely heterogeneous in nature, further long-term clinical observational study is needed to characterize potential late complications of ALF and improve follow-up care of survivors.

The use of PPIs is an independent protective factor of survival of ALF patients in multivariate analysis. Despite the lack of firm evidences, acid suppression by a PPI is recommended to prevent upper GI bleeding in intubated ALF patients or those in the ICU.^6^ By inference, proton pump inhibitors are likely to contribute to decreased incidence of significant upper gastrointestinal bleeding in patients with ALF.^5,38^ Peptic ulcer disease, in current study, is also an independent risk factor for intrahospital hemorrhage. Critical illness, such as respiratory failure and renal failure requiring renal replacement therapy, may also increase the risk of stress ulcer bleeding.^39^ To date, this is the first study to show the survival benefit of using PPIs in patients with ALF on their index admission. This study provides positive evidence for recommending the use of PPIs in ALF patients.

LT is considered as a lifesaving procedure for patients with ALF, but is not popular as a timely treatment option in this cohort. It may be due to the rapid course of ALF and the limited organ source. Artificial liver support with plasmapheresis and hemodialysis/hemofiltration plays a bridging role while a donor liver or the regeneration of the native liver is being awaited.^31^ Earlier studies report mortality rates near 85% before transplantation.^41^ However, in the posttransplantation era, 1-year survival rates are estimated to be 60% to 80%.^2,10,40,41^

During the study period, there were 88 liver transplant procedures (69 living donors and 19 deceased donors) in the National Taiwan University Hospital. Among them, 2 deceased-donor LTs were performed for ALF patients. The study cohort was a random sampling of 1 out of 23 million population of Taiwan. Thus, it is representative of the general, but not the transplant center, population. However, early recognition, prompt referral, and living donors in areas of low organ donation rates may save more lives of patients with ALF.^1,4,5,9,28,31^

This study has some limitations. Although the target cohort has been approached by utilizing multiple criteria, including the diagnosis, prescription of medications, and laboratory tests, the insurance reimbursement database has a built-in shortage of no information of laboratory data, radiographic findings, and medications not covered by insurance (ie, over-the-counter drugs). The frequency of laboratory testing and the statistics about hospital stay and ICU admission could be biased by the judgment of the attending physicians, but generally it is reasonable that more frequent laboratory testing and longer hospital or ICU stay would be expected in more critically ill patients. Nevertheless, it is very difficult to validate the diagnosis of ALF and the cause–effect relationship. Furthermore, patients with chronic liver disease may have been included if the disease has not been established within 3 years prior to the index admission.^5^ Therefore, using the 218 sample cases as the numerator to calculate the incidence of ALF may have some bias. This may also occur in previous studies and may be a reason for the heterogeneous clinical characteristics.^5,42–44^ However, it is less likely that a slowly progressed or even stable underlying liver disease, which requires no medical help within recent 3 years will rapidly deteriorate and result in liver failure without acute and new hepatic insult.

In summary, the incidence of ALF increases with age in Taiwan. Viral infection is the major etiology. Mortality rate is about 40% within 3 months and half of the survivors have concomitant liver cirrhosis. Patients with malignancy and alcohol exposure have the worst prognosis. Use of PPIs has a protective effect. LT for ALF is not highly utilized in Taiwan and early referral to a transplant center is recommended.

## ACKNOWLEDGMENTS

The authors thank the National Health Research Institute of Taiwan for providing the NHIRD.
