# Nationwide Longitudinal Analysis of Acute Liver Failure in Taiwan: Erratum

**DOI:** 10.1097/MD.0000000000008577

**Published:** 2017-11-03

In the article, “Nationwide longitudinal analysis of acute liver failure in Taiwan”,^[1]^ which appeared in Volume 93, Issue 4 of *Medicine*, Dr. Chih-Hsin Lee's affiliation should have appeared as Division of Pulmonary Medicine, Department of Internal Medicine, Wanfang Hospital and Division of Pulmonary Medicine, Department of Internal Medicine, School of Medicine, College of Medicine, Taipei Medical University, Taipei, Taiwan.

## References

[1] Ho C-M Lee C-H Wang J-Y Nationwide Longitudinal Analysis of Acute Liver Failure in Taiwan. *Medicine*. 2014 93:e35. 25068951 10.1097/MD.0000000000000035 PMC4602422

